# Significance of Affection Changes during Pregnancy: Intimacy, Passion, and Commitment

**DOI:** 10.3390/ijerph16132254

**Published:** 2019-06-26

**Authors:** Francisco Javier Fernández-Carrasco, Urbano González-Mey, Luciano Rodríguez-Díaz, Juana María Vázquez-Lara, Juan Gómez-Salgado, Tesifón Parrón-Carreño

**Affiliations:** 1 Department of Gynecology and Obstetrics, Hospiten Group, Hospiten Estepona, 29680 Estepona, Málaga, Spain; 2 Department of Surgery, Hospital Punta de Europa, 11207 Algeciras, Cádiz, Spain; 3 Department of Gynecology and Obstetrics, Hospital Universitario de Ceuta, Midwifery Teaching Unit of Ceuta, Spain, University of Granada, 51003 Ceuta, Spain; 4 Department of Sociology, Social Work, and Public Health, University of Huelva, 21007 Huelva, Spain; 5 Safety and Health Postgraduate Program, Espíritu Santo University, 092301 Samborondón, Guayaquil, Ecuador; 6 School of Health Sciences, University of Almeria, 04120 Almeria, Spain; 7 Territorial Delegation of Equality, Health and Social Policies, Health Delegation of Almeria, Junta de Andalucía, 04003 Almeria, Spain

**Keywords:** pregnancy, love, intimacy, passion, commitment, Sternberg

## Abstract

The complex process of gestation involves significant biological, psychological, and social changes for both women and men looking toward the same direction. The aim of this study was to analyze changes occurring in affective health between the members of a couple during pregnancy. For this, a cross sectional descriptive study using Sternberg’s questionnaire based on his Triangular Theory of Love (intimacy, passion, and commitment) was implemented. A total of 180 couples participated in the study. Data were collected four times during pregnancy: at the beginning, during the first trimester, the second trimester, and during the third trimester. The level of intimacy was higher at the beginning of pregnancy (123.93 ± 9.67), the same as the level of passion (119 ± 9.83). The commitment score in women was, in general, higher than in men. The commitment score in men increased at the beginning of the third trimester (124.31 ± 7.72). Statistically significant differences between the sexes were found for the level of commitment at the beginning of the pregnancy (*p* = 0.001) and at the third trimester (*p* = 0.008), these scores being higher for women than for men. No significant differences between men and women were found for the remaining components of the triangle. During pregnancy, no significant changes were found regarding overall affection throughout the entire period.

## 1. Introduction

Antoine de Saint-Exupéry said that ‘love does not consist in gazing at each other but in looking outward together in the same direction’ [1]. Pregnancy is closely linked to this approach to affection. Undoubtedly, the wonderful and complex process of gestation involves significant biological, psychological, and social changes for both women and men [2], particularly during the postpartum period [3]. Changes in the psychobiology sphere occurring during pregnancy can lead to sexual dysfunctions in women in this stage of their lives as well as in men [4,5,6].

The concept of love is difficult to define; therefore, it is difficult to measure. One of the first approaches to its conceptualization was developed by Robert J. Sternberg with his Triangular Theory of Love, where he suggested that the affective relationship could be understood in terms of three components: intimacy, passion, and commitment [7]. 

Intimacy refers to feelings of closeness, connectedness, and bondedness in loving relationships. Therefore, intimacy includes those feelings that lead essentially to the experience of warmth in a loving relationship. Passion refers to the impulses that lead to romance, physical attraction, sexual consummation, and the related phenomena in loving relationships. The passion component includes sources of motivation and other forms of arousal that lead to the experience of passion in a loving relationship. Commitment refers to the decision of loving each other, and, in the long-term, to maintaining the attachment.

Several studies on sexuality during pregnancy have been conducted. Von Sydow analyzed 59 studies on sexuality during pregnancy published between 1950 and 1996 and found changes in sexual interest and activity during pregnancy when compared to before pregnancy [8]. These studies focused on the coital activity of pregnant women and provided scarce or absent information about men as well as non-coital sexual activity and feelings. The studies conducted by Brtnicka et al. also focused on the interest in sex and the sexual activity of pregnant women [9]. Similarly, Sacomori and Cardoso studied sexual practices in pregnant Brazilian women using questionnaires [10]. Nevertheless, there have been some studies that have approached affection [11]. Others have even approached the feelings, love, and affection of pregnant women, which considered that these feelings strengthened self-satisfaction (satisfaction with themselves) and promoted life continuity, symbolized by pregnancy [12].

In a similar way, studies focusing on men had only explored the sexual behaviors and beliefs related to the convenience of sexual intercourse during pregnancy, the frequency of sex, and even the convenience of having an extra-marital partner during this period [13,14]. LaRossa and Sapién and Córdoba conducted qualitative studies about the sexual activity of men during pregnancy [15,16]. However, few researchers have approached the measure of this issue using the tool described by Sternberg and Grajek [17], as modified by Yela [18], or their own tool such as Rusbult [19], but none have focused on pregnancy and no one has assessed the behavior of triadic love during pregnancy.

## 2. Materials and Methods 

The present study parts from the meaning of an affective relationship for the members of different couples and analyzes the affective changes they experience during the entire pregnancy. A cross-sectional descriptive study was conducted. The study population consisted of pregnant women and their male partners who visited the Hospiten Estepona Health Centre in Estepona (Spain) for their first prenatal care visit from January 2017 to December 2018.

The sample size was considered sufficiently high when it determined differences of up to nine units between the paired means (i.e., the means between one trimester and the following one), a mean SD of both means of 30, and a significance level of 0.05, two-tailed contrast, and power of 0.80. This sample was randomly chosen among all women attending the healthcare center for prenatal care who met the inclusion criteria and whose partners also agreed to participate in the study. 

The inclusion criteria were as follows:

- Pregnant women recruited since the first prenatal care visit;
- Men who were partners of these women; and
- Both of them held Spanish nationality.

The exclusion criteria included:

- Pregnant women who had not been recruited from the beginning of their pregnancies;
- Women who developed any kind of disease during pregnancy; and
- Women whose partners rejected enrolling in the study.

A total of 147 were initially recruited and 39 were excluded because their partners did not wish to participate in the study. Finally, 108 couples were included in the study.

The study analyzed sociodemographic variables such as sex, age, marital status, schooling level, number of children, number of miscarriages, and level of commitment. Data were collected using Sternberg’s love scale, and sociodemographic variables were collected using an ad hoc questionnaire. Sternberg’s love scale was distributed among the participants four times: at the first prenatal care visit, which was before week nine of pregnancy (beginning of pregnancy); at week 12 of pregnancy (first trimester); between weeks 20 and 24 of pregnancy (second trimester); and after week 32 of pregnancy (third trimester).

Sternberg’s Love Scale was validated in Spain by Carreño and Serrano [20]. The total Cronbach’s alpha was 0.96. Cronbach’s alpha values were 0.94, 0.88, and 0.93 for the intimacy, passion and commitment subscales, respectively.

During the first visit, participants were informed about the purpose and the development of the study. A written informed consent was signed by both members of the couple prior to participation. Data confidentiality was guaranteed as the information was recorded anonymously. The study was given consent from the directors and approved by the Ethics Committee of Research of the Hospiten Estepona Health Centre.

A statistical analysis was performed using the software SPSS version 23 (Windows, Chicago, IL, USA). Frequencies and percentages were determined for the qualitative variables. Means and standard deviations (SD) were determined for the quantitative variables. Pearson’s correlation coefficient, the Mann–Whitney U test, and the Kruskal–Wallis test were used for the bivariate analysis. A *p* < 0.05 was considered significant. 

## 3. Results

The mean values, standard deviations, maximum, and minimum values are shown in Table 1.

With regard to marital status, Table 2 shows that most of the participants were married, almost 70% of the sample.

Table 3 shows that most of the participants in the study were graduates or postgraduates.

The bivariate analysis showed statistically significant differences regarding the age variable, which was slightly lower in the women than in the men (Table 4).

When performing the repeated measures analysis with the test totals (ANOVA), statistically significant differences were found both when analyzing the men and women combined and individually (*p* < 0.001). The Bonferroni test allows for the comparison of the means of the t levels of a factor after having rejected the null hypothesis (Ho) of the equality of means using the ANOVA technique. This test adjusts the level of significance in relation to the number of statistical tests performed simultaneously on a dataset. It is a test of multiple comparisons to avoid accumulating an error of 5% in each comparison pair. The Bonferroni test is based on the creation of a threshold above which the difference between the two means will be significant and below which this difference will not be statistically significant. When performing the post hoc Bonferroni test, significative differences were found between the second trimester and the rest (*p* < 0.001), and for both the combined analysis and the one performed on women. On the other hand, as for men, a difference was found between the second trimester and the first and fourth ones (*p* < 0.001) (Table 5).

**Figure 1:**
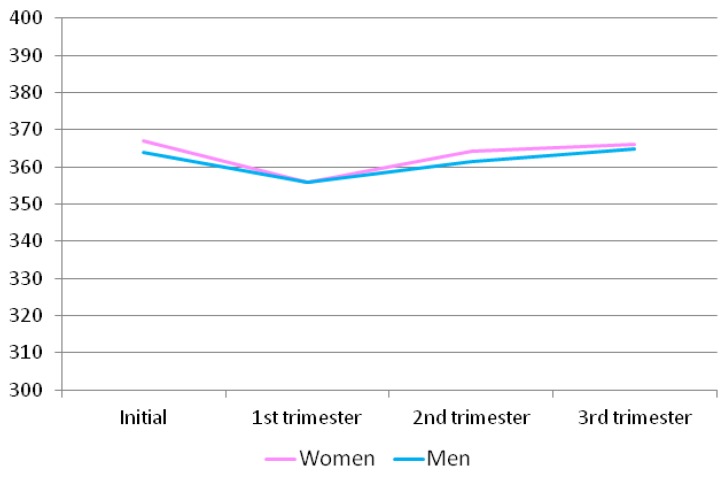
Total test scores of the men and women at different time points throughout pregnancy.

With regards to the total test scores of the men and women at different time points throughout pregnancy, results are shown in Figure 1. Regarding the comparison by sex of the test domain scores and the total scores, statistically significant differences were only found regarding the initial commitment, with women obtaining higher scores than men (123.61 vs. 120.28). Likewise, statistically significant differences were found regarding commitment in the third trimester, with women also obtaining higher scores than men. No differences were found for the other domains or any of the four determinations of each remaining domain (Table 6).

By comparing the test scores with the schooling level, statistically significant differences were only found for commitment, with the highest scores found in uneducated participants or those with primary school education, followed by graduates or postgraduates. The lowest score was found in participants who had completed secondary/high school or technical education (Table 7).

When comparing all of the test components, the age and the number of children showed a positive correlation with a high and statistically significant Pearson’s coefficient. A significant correlation coefficient of 0.70 was found between intimacy and passion. Similarly, a significant correlation coefficient of 0.74 was found between intimacy and commitment. The correlation between intimacy and total score was higher than the other two correlations above-mentioned (0.90). A negative correlation was found between intimacy and age, but this value was not statistically significant. No significant correlation was found between intimacy and the number of children. As can be seen in Table 7, there was a positive and significative correlation between the three domains and the test total score as there was between commitment and the number of children 0.16 (*p* < 0.05).

Passion was significantly correlated with commitment (0.75) and with the total score (0.92). A negative correlation was found between passion and age, but this correlation was not significant. No significant correlation was found between passion and the number of children.

Commitment was significantly correlated with the total score (0.90). A negative correlation was found between commitment and age, but this correlation was not significant. No significant correlation was found between commitment and the number of children.

Total score was also negatively correlated with age and the number of children, but these correlations were not significant (Table 8).

**Figure 2:**
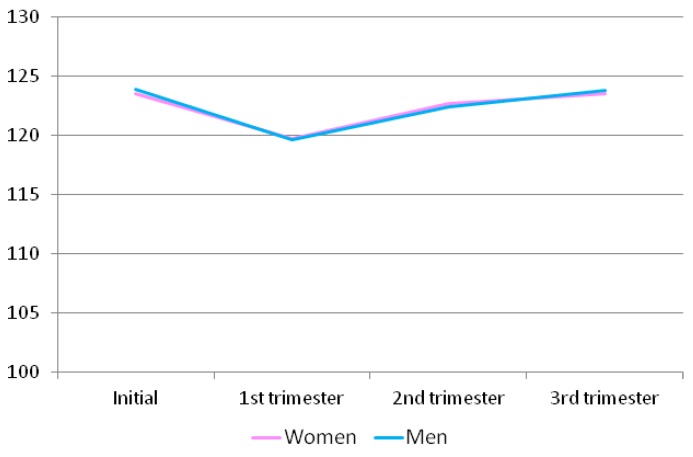
Intimacy scores for the men and women at different time points throughout pregnancy.

The intimacy domain scores (Table 9 and Figure 2) remained practically the same. Regarding the scores of the women and men, these were higher at the beginning, decreased in the first trimester, and increased again in the second and third trimesters. Similarly, the same happened with the passion domain.

The post hoc Bonferroni test stablished a difference between the first trimester total scores and those of the other trimesters (*p* < 0.001), and between the second and the third trimesters (*p* = 0.026). Regarding women, the difference was established between the first trimester and the others (*p* < 0.001), and between the second and the third trimesters (*p* = 0.045). Regarding the Mann–Whitney U test, the difference was established between the first trimester and the others (*p* < 0.047).

**Figure 3:**
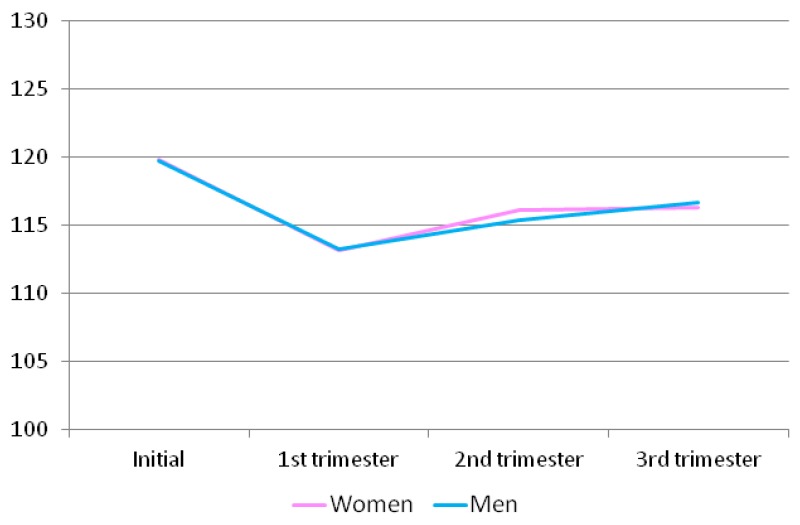
Passion scores for the men and women at different time points throughout pregnancy.

Regarding the passion domain (Table 10 and Figure 3), the highest score was found at the beginning of the process. Then, it critically decreased, reaching the lowest level in the first trimester and increased again during the second and the third trimesters, though did not reach the initial level. In the second trimester, the score was slightly higher in women than in men.

The post hoc Bonferroni test established a difference between the initial total and the first trimester, and then between the initial total and each of the other trimesters (*p* < 0.001). For women, this difference was established between the initial total and the first trimester and between the initial total and each of the other trimesters (*p* < 0.001). Regarding the Mann–Whitney U test, this difference was also established between the initial total and the first and rest of the trimesters (*p* < 0.005).

**Figure 4:**
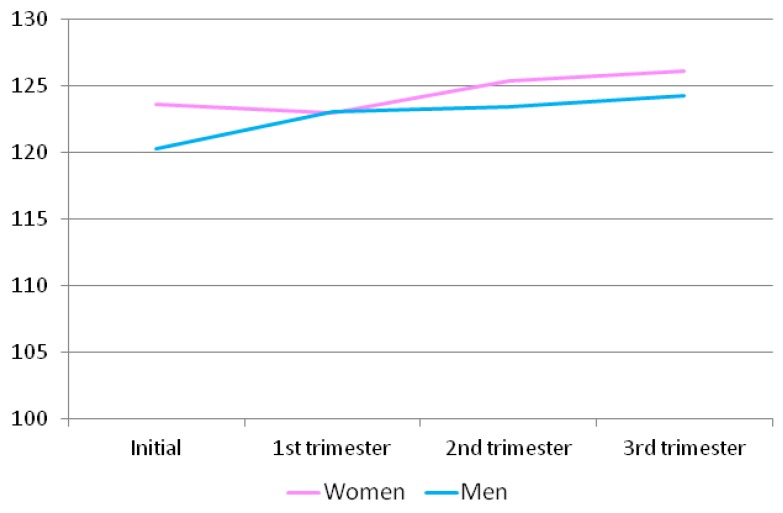
Commitment scores for the men and women at different time points throughout pregnancy.

Regarding the commitment domain, as can be seen in Table 11 and Figure 4, women always obtain higher scores than men, but the latter increase their scores from the beginning until the third trimester, while women show a slight decrease of these scores in the second trimester.

The post hoc Bonferroni test established a difference between the initial scores and those of the second and third trimesters (*p* < 0.004) and between the first and the second and third trimesters (*p* < 0.04). For women, this difference was established between the initial scores and those of the third trimester (*p* < 0.05) and between the first and the second and third trimesters (*p* < 0.001). Regarding the Mann–Whitney U test, this difference was established between the first and third trimester (*p* < 0.003).

Four linear mixed-effects regression models (LRM-ME) were developed for each dependent variable (questionnaire dimensions) that considered the randomized effect of the participants. Model I was null; model II was adjusted by the time variable; model III included the sex variable; and, eventually, model IV was adjusted by time and sex (only for those dependent variables whose independent variables were statistically significant in models II and III). The intercept variance shown in Table 12 indicates the individual variability.

Model II

An increase in time also increased intimacy by 0.279, significantly decreased passion by −0.72 (*p* < 0.001), and increased commitment by 1.13.

Model III

Being male decreased commitment by −1.74, without significantly affecting the intimacy and passion dimensions.

Model IV

An increase in time also increased commitment by 1.131, while the sex variable remained the same (*p* < 0.001). Being male decreased commitment by −1.741, while the time variable remained the same.

## 4. Discussion

Many studies using Sternberg’s Triangle Theory of Love have been published. However, so far, no one has investigated the importance of affective health during pregnancy as the sum of the three components (intimacy, passion, and commitment) proposed by this theory. This study aimed to elucidate the changes occurring in these parameters for each trimester throughout pregnancy. 

Intimacy decreased slightly during the first trimester. Then, intimacy slowly recovered during the second and third trimesters, reaching similar levels to those found at the beginning in both sexes. Our results are consistent with recent studies showing no differences between the sexes regarding intimacy [21,22]. In contrast, other studies described higher levels of intimacy for women than for men [23,24]. Nevertheless, Dindia and Allen, in a meta-analysis, reported that this difference was small [25]. Findings revealed a dip in intimacy and passion during the first trimester. This may occur because the woman in the first trimester may feel discomfort from pregnancy such as nausea, hypersensitivity, etc., which would make her less keen to be intimate or express the passion she would express if she felt completely well. In addition, there is a belief and fear that a miscarriage may occur as a result of sexual contact, thereby diminishing the expression of intimacy and passion.

The outcomes of this study are consistent with the results found by Sorokowski et al. [26], as we also found an unexpected negative correlation between the women’s intimacy scores and the number of children, although this correlation was not statistically significant. It is likely that women that need to look after more children have less time to spend with their partners, which explains this lower level of intimacy. Mothers can also satisfy their need for intimacy through close contact with their children. Previous studies have demonstrated that the transition to parenthood can interfere with the sexual life and partnership within the couple [27], and that marital relations and parent–child relationships are interrelated [28].

Regarding passion, our study is consistent with recent studies that have stated that passion does not differ between sexes [29]. However, our results showed that passion clearly decreased during the first trimester of pregnancy in both men and women. However, as pregnancy progressed, passion was gradually recovered.

Regarding commitment, and according to Tsirigotis et al., women showed a greater level of commitment than men under normal conditions [30]. This study is also consistent with our results. However, despite women having a higher level of commitment than their partners throughout the entire pregnancy, we found that as pregnancy progressed, commitment became stronger among men. Nevertheless, other studies suggest that there are no differences between men and women regarding commitment.

## 5. Conclusions

Love that we may have toward our partner is different throughout the relationship, and therefore, love can change during different stages and situations in our lives. 

Despite a significant change in the level of commitment (one of the three components) that was found during pregnancy, no significant changes were found for total affection throughout the entire period. During pregnancy, men increased their total level of commitment to their partner. At the end of pregnancy, men reached higher commitment levels than they had before gestation, whereas women maintained the same level throughout the entire pregnancy.

The results from this research may help in understanding the effect of pregnancy in the relationship of a couple. These could also help to identify situations of stress or lack of coping strategies. The results are also helpful in understanding the loving and caring environment that will welcome the baby.

## Figures and Tables

**Table 1:** Mean values of the quantitative variables studied.

Variables	*N*	Minimum	Maximum	Mean	S.D.
**Women’s age**	108	24	43	32.72	4.20
**Men’s age**	108	21	48	33.85	5.46
**Years of relationship**	216	2	20	6.77	3.28
**Number of children**	216	0	3	0.60	0.74
**Number of miscarriages**	216	0	2	0.11	0.34
**Intimacy**	216	98.00	135.00	122.43	8.57
**Passion**	216	90.00	133.75	116.30	10.66
**Commitment**	216	99.00	133.50	123.65	6.98
**Total Test**	216	99.00	133.25	120.79	7.92

**Table 2:** Distribution of marital status.

Marital Status	Frequency	Percentage
**Married**	195	69.6
**Single**	61	21.8
**Divorced**	24	8.6
**Total**	280	100.0

**Table 3:** Distribution of schooling level.

Schooling Level	Frequency	Percentage
**Uneducated or Primary School**	48	22.2
**High or Technical School**	75	34.7
**Graduate or Postgraduate**	93	43.1
**Total**	216	100.0

**Table 4:** Mean age based on sex.

Sex	*N*	Minimum	Maximum	Mean	S.D.	*p*-Value *
**Women**	108	24	43	32.72	4.20	<0.001 *
**Men**	108	21	48	33.85	5.46	

* Asterisks represent statistically significant differences between men and women (Mann–Whitney U test, *p*-value < 0.05).

**Table 5:** Initial and final total scores of the means of each trimester.

Scores		Mean	S.D.	*p*-Value *
TOTAL	Initial	365.47	1.69	0.001
	1st Trimester	355.86	1.88	
	2nd Trimester	362.77	1.85	
	3rd Trimester	365.46	1.74	
WOMEN	Initial	367.00	2.20	0.001
	1st Trimester	355.80	2.88	
	2nd Trimester	364.25	2.56	
	3rd Trimester	366.08	2.65	
MEN	Initial	363.95	2.58	0.001
	1st Trimester	355.91	2.43	
	2nd Trimester	361.30	2.67	
	3rd Trimester	364.84	2.26	

* Repeated measures ANOVA.

**Table 6:** Comparison by sex of the test domain scores and the total scores.

Domains	Sex	*N*	Mean	S.D.	*p*-Value *
Initial Intimacy	Female	108	123.58	8.48	0.84
	Male	108	123.93	9.67	
1st Trimester Intimacy	Female	108	119.72	11.48	0.51
	Male	108	119.65	10.58	
2nd Trimester Intimacy	Female	108	122.75	9.39	0.96
	Male	108	122.47	10.28	
3rd Trimester Intimacy	Female	108	123.58	9.09	0.77
	Male	108	123.83	7.56	
Initial Passion	Female	108	119.81	9.38	0.66
	Male	108	119.75	11.22	
1st Trimester Passion	Female	108	113.14	12.24	0.85
	Male	108	113.21	12.07	
2nd Trimester Passion	Female	108	116.14	11.53	0.96
	Male	108	115.36	12.47	
3rd Trimester Passion	Female	108	116.33	11.25	0.54
	Male	108	116.69	12.34	
Initial Commitment	Female	108	123.61	7.42	0.001
	Male	108	120.28	8.97	
1st Trimester Commitment	Female	108	122.94	9.39	0.35
	Male	108	123.06	7.31	
2nd Trimester Commitment	Female	108	125.36	8.33	0.17
	Male	108	123.47	8.91	
3rd Trimester Commitment	Female	108	126.17	9.38	0.008
	Male	108	124.31	7.72	
Initial Total	Female	108	367.00	22.89	0.28
	Male	108	363.95	26.90	
1st Trimester Total	Female	108	355.80	29.93	0.62
	Male	108	355.91	25.28	
2nd Trimester Total	Female	108	364.25	26.67	0.47
	Male	108	361.30	27.76	
3rd Trimester Total	Female	108	366.08	27.59	0.52
	Male	108	364.84	23.57	
Total Mean	Female	108	121.09	8.51	0.39
	Male	108	120.50	7.31	

* Mann–Whitney U test.

**Table 7:** The total mean scores of each domain and the total sum test according to the level of education.

Domains	Schooling	*N*	Mean	S.D.	*p*-Value *
**Intimacy**	Uneducated or Primary School	48	124.26	7.18	0.32
	High or Technical School	75	121.24	9.93	
	Graduate or Postgraduate	93	122.46	7.94	
**Passion**	Uneducated or Primary School	48	117.52	9.77	0.15
	High or Technical School	75	117.28	11.14	
	Graduate or Postgraduate	93	114.88	10.64	
**Commitment**	Uneducated or Primary School	48	126.13	4.45	<0.001
	High or Technical School	75	121.28	7.23	
	Graduate or Postgraduate	93	124.28	7.33	
**Total**	Uneducated or Primary School	48	122.63	5.86	0.54
	High or Technical School	75	119.93	9.06	
	Graduate or Postgraduate	93	120.54	7.78	

* Kruskal–Wallis test.

**Table 8:** Correlations between the total components’ scores, age, and number of children.

Components		Passion	Commitment	Total	Age	Number of Children
**Intimacy**	Correl. Coef.	0.70	0.74	0.90	−0.073	0.09
	Sig. (Bilateral)	<0.001	<0.001	<0.001	0.28	0.15
**Passion**	Correl. Coef.		0.75	0.92	−0.065	0.03
	Sig. (Bilateral)		<0.001 *	<0.001	0.34	0.58
**Commitment**	Correl. Coef.			0.90	−0.031	0.16
	Sig. (Bilateral)			<0.001	0.64	0.02
**Total**	Correl. Coef.				−0.06	0.10
	Sig. (Bilateral)				0.34	0.14
**Age**	Correl. Coef.					0.30
	Sig. (Bilateral)					<0.001

* Asterisks represent statistically significant differences between groups (Pearson’s correlation coefficient *p* value < 0.05).

**Table 9:** Initial and final intimacy means for each trimester.

Intimacy		Mean	S.D.	*p*-Value *
Total	Initial	123.75	0.61	0.001
	1st Trimester	119.68	0.75	
	2nd Trimester	122.61	0.66	
	3rd Trimester	123.70	0.56	
Women	Initial	123.58	0.81	0.001
	1st Trimester	119.72	1.10	
	2nd Trimester	122.75	0.90	
	3rd Trimester	123.58	0.87	
Men	Initial	123.92	0.93	0.001
	1st Trimester	119.64	1.01	
	2nd Trimester	122.47	0.99	
	3rd Trimester	123.83	0.72	

* Repeated measures ANOVA.

**Table 10:** Initial and final passion means for each trimester.

Passion		Mean	S.D.	*p*-Value *
Total	Initial	119.77	0.70	0.001
	1st Trimester	113.17	0.82	
	2nd Trimester	115.75	0.816	
	3rd Trimester	116.51	0.802	
Women	Initial	119.80	0.903	0.001
	1st Trimester	113.13	1.178	
	2nd Trimester	116.13	1.110	
	3rd Trimester	116.33	1.083	
Men	Initial	119.7	1.08	0.001
	1st Trimester	113.21	1.16	
	2nd Trimester	115.36	1.20	
	3rd Trimester	116.69	1.18	

* Repeated measures ANOVA.

**Table 11:** Initial and final Commitment means for each trimester.

Commitment	Period	Mean	S.D.	*p*-Value *
Total	Initial	121.94	0.57	0.001
	1st Trimester	123.00	0.57	
	2nd Trimester	124.41	0.58	
	3rd Trimester	125.24	0.58	
Women	Initial	123.61	0.71	0.001
	1st Trimester	122.94	0.90	
	2nd Trimester	125.36	0.80	
	3rd Trimester	126.16	0.90	
Men	Initial	120.27	0.86	0.001
	1st Trimester	123.05	0.70	
	2nd Trimester	123.47	0.85	
	3rd Trimester	124.31	0.74	

* Repeated measures ANOVA.

**Table 12:** Linear mixed-effects regression model (LRM-ME).

Intimacy	Model I	Model II	Model III	Model IV
*Fixed effects, coefficient (S.D.)*				
Intercept	122.440 (0.528)	122.023 (0.631)	122.410 (0.822)	
Time		0.279 (0.163) **		
Sex			0.060 (0.164)	
*Random effects, variance (S.D.)*				
Intercept	65.896 (7.048)	65.928 (7.048)	65.895 (7.048)	
Residual	28.913 (1.606)	28.783 (1.599)	28.913 (1.606)	
**Passion**				
*Fixed effects, coefficient (S.D.)*				
Intercept	116.304 (0.724)	117.387 (0.771)	116.354 (1.024)	
Time		−0.722 (0.176) *		
Sex			−0.100 (1.448)	
*Random effects, variance (S.D.)*				
Intercept	104.590 (10.904)	104.807 (10.903)	104.588 (10.904)	
Residual	34.462 (1.915)	33.594 (1.866)	34.461 (1.915)	
**Commitment**				
*Fixed effects, coefficient (S.D.)*				
Intercept	123.651 (0.474)	121.955 (0.539)	124.521 (0.665)	122.825 (0.713)
Time		1.131 (0.171) *		1.131 (0.171) *
Sex			−1.741 (0.941) **	−1.741 (0.940) **
*Random effects, variance (S.D.)*				
Intercept	40.137 (4.697)	40.670 (4.694)	39.380 (4.624)	39.912 (4.621)
Residual	33.715 (1.873)	31.585 (1.755)	33.715 (1.873)	31.585 (1.755)

Model I: Null model; Model II: Includes the Time variable; Model III: Includes the Sex variable; Model IV: Includes both Sex and Time. * *p* value < 0.001 ** *p* value ≤ 0.10. S.D.: standard deviation
